# The delay effect on outcome evaluation: results from an event-related potential study

**DOI:** 10.3389/fnhum.2013.00748

**Published:** 2013-11-13

**Authors:** Chen Qu, Yunyun Huang, Yuru Wang, Yu-Xia Huang

**Affiliations:** ^1^Psychology Research Center, South China Normal UniversityGuangzhou, China; ^2^State Key Laboratory of Cognitive Neuroscience and Learning, and International Data Group/McGovern Institute for Brain Research, Beijing Normal UniversityBeijing, China; ^3^Center for Collaboration and Innovation in Brain and Learning Sciences, Beijing Normal UniversityBeijing, China

**Keywords:** decision-making, temporal discounting, reward, event-related potential, feedback-related negativity

## Abstract

Behavioral studies demonstrate that the timing of receiving gains or losses affects decision-making, a phenomenon known as temporal discounting, as participants are inclined to prefer immediate rewards over delayed ones and vice versa for losses. The present study used the event-related potential technique with a simple gambling task to investigate how delayed rewards and losses affected the brain activity in outcome evaluations made by 20 young adults. Statistical analysis revealed a larger feedback-related negativity (FRN) effect between loss and gain following immediate outcomes than following future outcomes. In addition, delay impacted FRN only in gain conditions, with delayed winning eliciting a more negative FRN than immediate winning. These results suggest that temporal discounting and sign effect could be encoded in the FRN in the early stage of outcome evaluation.

## INTRODUCTION

Time is an important dimension when assessing the value of a reward in a decision-making situation because when delivery of a reward is delayed, an individual’s valuation of a future reward declines (Mazur, 1987). This phenomenon is generally referred to as temporal discounting (Samuelson, 1937; Ainslie, 1975). As temporal discounting is ubiquitous in daily decision-making and impairments in temporal discounting characterize a range of psychiatric conditions (i.e., substance abuse, addiction and attention-deficit hyperactivity disorder), this topic has shown rapid progress in the past few years. Brain research has provided insight into the neural mechanisms underlying temporal discounting. McClure et al. (2004) proposed that two separate neural systems value immediate and delayed rewards. Specifically, a limbic system (β) is thought to place special weight on immediate rewards, whereas a more cognitive, prefrontal-cortex-based system (δ) is more involved in patient choices. However, single valuation account holds that the values of both immediate and delayed rewards are represented in a unitary system encompassing the ventral striatum, medial prefrontal cortex, and posterior cingulate cortex (Kable and Glimcher, 2007, 2010). Nevertheless, in the self-control account, values are assumed to be represented in structures such as the ventromedial prefrontal cortex (vmPFC) but are subject to top-down modulation by prefrontal control regions such as the lateral PFC (Hare et al., 2009; Figner et al., 2010).

However, the above research have been predominantly concerned with discounting future gains rather than on losses, although losses may seem as important as gains, as many of the most-discussed real-world phenomena relating to intertemporal choice involve aversive outcomes (Harris, 2012). Behavioral evidence suggests that people usually discount delayed losses less steeply than delayed gains (Thaler, 1981; Loewenstein, 1987; MacKeigan et al., 1993; Read, 2004). This phenomenon, termed the sign effect (Loewenstein, 1987), is rarely tested with electrophysiology. Furthermore, while fMRI studies have provided abundant evidence of the brain mechanism of temporal discounting, the time course of cortical activation has not been studied precisely.

The event-related potential (ERP) techniques with high temporal resolution have provided critical temporal information for the neural correlates of temporal discounting. However, it remains unclear whether temporal information and valence could be encoded and integrated in the process of outcome evaluation. Feedback-related negativity (FRN) is an important ERP component implicated in reward processing (Gehring and Willoughby, 2002). The FRN, generated by the anterior cingulate cortex (ACC; Gehring and Willoughby, 2002; Holroyd and Coles, 2002), has been conceptualized as a negative deflection around 250 ms post-onset of the feedback stimulus (e.g., Holroyd and Coles, 2002; Hajcak et al., 2006). The FRN is more pronounced for negative feedback associated with unfavorable outcome, such as incorrect response or monetary loss, than for positive feedback (Miltner et al., 1997; Gehring and Willoughby, 2002; Yeung and Sanfey, 2004; Holroyd et al., 2006; Goyer et al., 2008). Weinberg et al. (2012) examined the effect of feedback delay on reward processing, but in their study delay referred to delayed feedback following prior action after a short delay of 6 s, while many of human choices only pay off after months or even years. Blackburn et al. (2012) examined electrophysiological correlates involved in the detection and evaluation of immediate and delayed monetary outcomes. However, in their study participants processed only temporal information in outcome evaluation, while in most environments temporal discounting involves both valence and temporal information. Another study, mainly focused on individual difference, found that FRN classifies outcomes in a binary manner, with immediate non-reward, delayed non-reward, and delayed reward all perceived as unfavorable outcomes (Cherniawsky and Holroyd, 2013). Nevertheless, because they used a non-reward (1 penny) as negative feedback, it is still unclear whether FRN could distinguish losses with different time delays.

The present study comprehensively investigated the neural basis of temporal discounting to verify whether temporal information and valence could be integrated and encoded in the FRN and P300. Using ERP technique, we utilized a relatively straightforward gambling paradigm in which participants attempted to guess which of two pictures hid a monetary reward. Feedback indicating whether participants gain or lose money was presented after each response. There were four kinds of outcome feedback: gain 10 RMB immediately, gain 10 RMB a month later, lose 10 RMB immediately, and lose 10 RMB a month later (RMB, the Chinese currency, is the abbreviation of Ren Min Bi, and rough estimated value for 10 RMB is 1.634 dollars). According to previous research on temporal discounting and the sign effect, the subjective value of immediate gains was larger than that of delayed gains, while for losses, this difference tended to be smaller. Therefore, we hypothesized that FRN would be sensitive to valence and temporal delay. The difference between gain and loss in the time range of the FRN would be substantial for immediate rewards, but the difference would be reduced for delayed rewards. In addition, we examined whether the P300, the most positive peak in the 250–450 ms time window post-onset of feedback, would be impacted by delay.

## MATERIALS AND METHOD

### PARTICIPANTS

Twenty undergraduates from Beijing Normal University were recruited online. The mean age of the participants was 21 ± 1.63 years, ranging between 19 and 25 years. All participants were right handed and had normal or corrected-to-normal vision, and had no history of neurological, psychiatric, or cognitive disorders. Informed consent was obtained from each participant. This study was approved by the Ethics Committee of State Key Laboratory of Cognitive Neuroscience and Learning at Beijing Normal University.

### STIMULI AND PROCEDURES

The experiment had a two (temporal delay: immediate vs. delayed) by two (outcome valence: gain vs. loss) factorial design. Four experimental conditions were composed of four types of outcome feedback: gain 10 RMB now, gain 10 RMB a month later, lose 10 RMB now, and lose 10 RMB a month later. Each feedback consisted of a photo of the 10 RMB (The photo was either colored or in black-and-white, indicating gain or loss of money respectively), with the time of reward delivery written below: “Now” or “1 month”.

**FIGURE 1:**
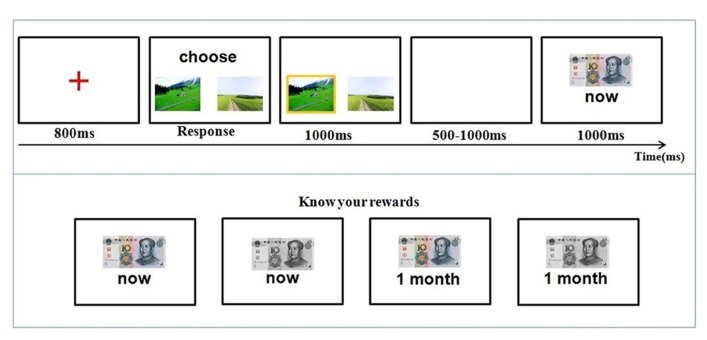
**Time course of stimulus presentation in the gambling task.** Participants were informed about the four possible outcomes: gain 10 RMB now, gain 10 RMB a month later, lose 10 RMB now, and lose 10 RMB a month later (bottom panel). They then chose one of two cards by pressing the corresponding button. At the end of each trial, participants were informed about their outcome (top panel).

The time course of a trial is illustrated in **Figure 1**. At the beginning of each trial, participants were first presented with a red cross for 800 ms at the center of the screen, then two photos of landscapes were presented, and participants were required to select one of them by pressing corresponding keys. The two pictures were presented on the screen until the participant made a choice. They needed to press “F” if they chose the picture on the left and “J” if they chose the picture on the right. The selected photo was highlighted by a yellow border. After a random time interval (500–1000 ms), the feedback of winning or losing was shown for 1000 ms. The next trial began 1 s after the offset of the feedback.

Before the ERP recordings, participants were provided with verbal instructions and a training session to familiarize with the procedure. The formal experiment consisted of four blocks of 60 trials per block. On each trial, four options were available (i.e., gain money now, lose money now, gain money later, or lose money later). Between the blocks, participants were provided with a self-timed rest period. They were told that they could adopt whatever strategies they wanted to use to maximize their rewards. Unbeknownst to the participants, feedback was predetermined and randomized, with each kind of feedback appearing in equivalent numbers of 60 trials. Participants were assured that they would get 40 RMB as basic payment (20 RMB for today and 20 RMB for 1 month later) and that additional monetary reward would be paid according to their performance, with the immediate rewards to be delivered following completion of the experiment, and the future rewards to be sent via a check from the university 1 month later; however, in the end all participants were paid 50 RMB (about $8.17) immediately in cash.

### ERP RECORDING AND ANALYSIS

EEGs were recorded from 32 scalp sites using tin electrodes mounted in an elastic cap (NeuroScan Inc., USA) according to the international 10–20 system. The vertical electrooculogram (VEOGs) were recorded from electrodes located above and below the left eye. The horizontal EOG (HEOG) was recorded by electrodes placed 1.5 cm lateral to the left and right external canthi. All EEGs and EOGs were referenced online to the left mastoid and off-line algebraic re-referenced to the average of the left and right mastoids. A ground electrode was placed on the medial frontal aspect. The interelectrode impedances were maintained below 5 kΩ. The EEG and EOG were amplified (bandpass 0.05–100 Hz) and digitized online with a sampling frequency of 500 Hz.

The EEG data were preprocessed with Brain Vision Analyzer software. Ocular artifacts were corrected with an eye-movement correction algorithm, which employs a regression analysis in combination with artifact averaging (Gratton et al., 1983). A 1000 ms epoch of data, extending from 200 ms prior to 800 ms following the onset of each feedback stimulus, was extracted from the continuous data file for analysis, with the 200 ms pre-stimulus EEG activity used for baseline correction. All trials in which EEG voltages exceeded a threshold of ±90 μV during the recoding epoch were excluded from analysis. The EEG data were low-pass filtered using a 20 Hz low-pass (24 dB octave roll off), and were baseline-corrected by subtracting the average activity of that electrode during the baseline period from each sample.

**FIGURE 2:**
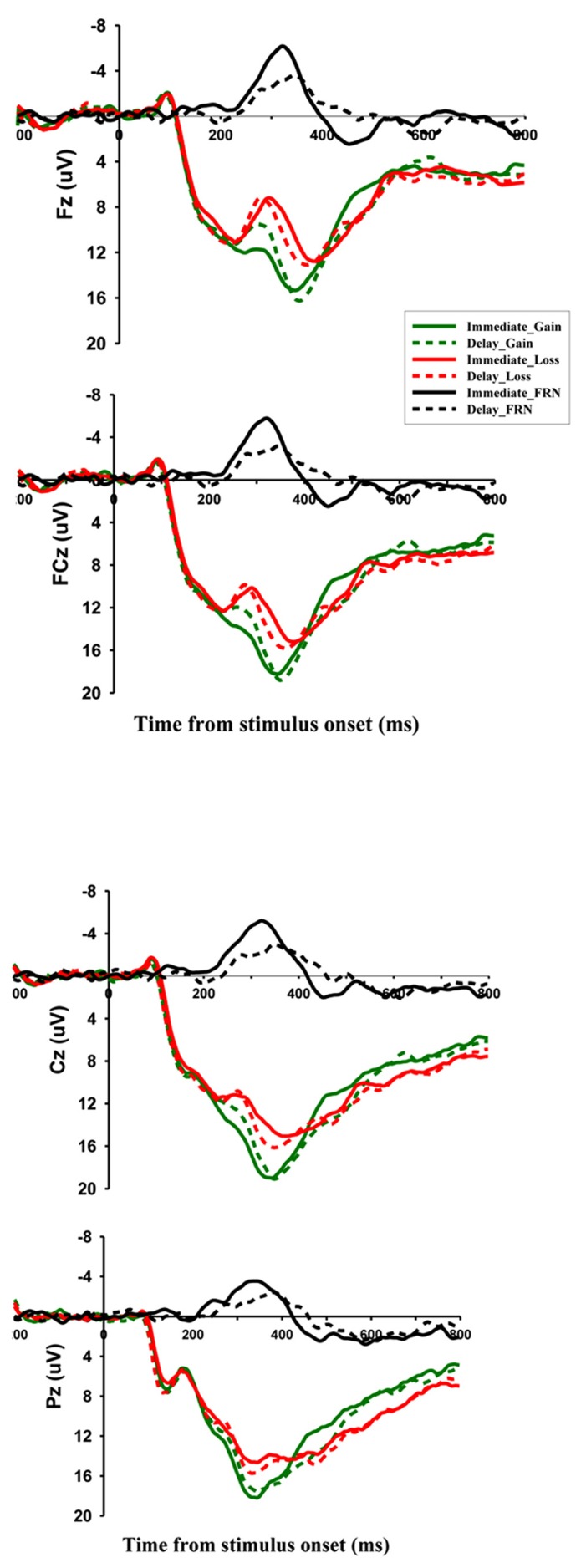
**Grand-averaged ERPs and difference waves for immediate rewards and delayed rewards at Fz, FCz, Cz, and Pz.** Difference waves were created by subtracting the feedback-evoked ERP associated with negative feedback from the ERP associated with positive feedback. The solid black line represents difference waves for immediate rewards, while the dotted black line represents difference waves for delayed rewards.

**FIGURE 3:**
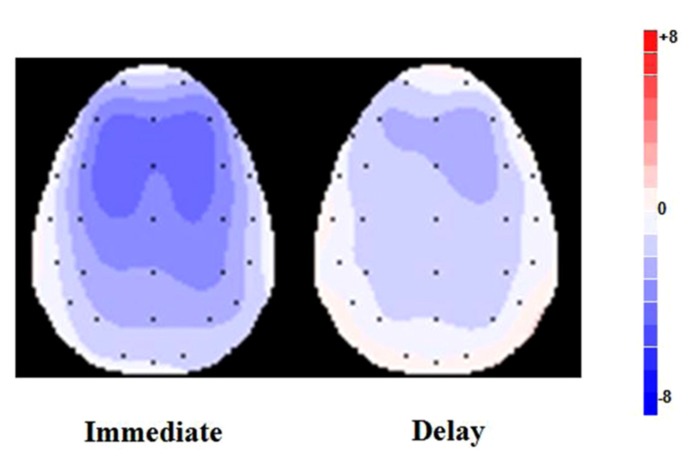
**Scalp topographies of the difference waves between ERP responses to the loss vs. gain outcomes averaged for FRN in the time range of 230–330 ms**.

The ERP components that were analyzed were FRN and P300. Time windows were selected for analysis based on visual inspection of the waveforms and their scalp distributions (**Figures 2 and 3**). For the FRN, we measured the mean amplitude in the time window of 230–330 ms post-onset of the feedback. To minimize the effects of overlap between ERP components, most notably the P3, we created difference waves by subtracting ERPs elicited by loss feedback from ERPs associated with gain feedback and used the mean values of the difference waves in the 230–430 ms time window as measures of the FRN effect (Holroyd and Krigolson, 2007). For the P300, we took the peak amplitudes in the time window of 250–450 ms. We focused on the four electrode locations in the midline (Fz, FCz, Cz, and Pz), where these components had been most pronounced in previous studies. Separate repeated measures analyzes of variance (ANOVAs) were conducted for the two potentials with three within-participant factors: electrode location (Fz, FCz, Cz, and Pz), temporal delay (immediate vs. delayed) and outcome valance (gain vs. loss). In all analyzes, the Greenhouse-Geisser correction was applied when the assumption of sphericity was violated.

## RESULTS

### FEEDBACK-RELATED NEGATIVITY

**Figures 2 and 3** indicate that, in accordance with previous studies reporting FRN, negative feedback elicited a negative-going waveform that reached its maximum over frontocentral scalp Positions. For the FRN amplitude, a three-way repeated measures ANOVA with factors of feedback valence (loss vs. gain), temporal delay (delayed vs. immediate), and electrode location (Fz, FCz, Cz, and Pz) revealed a significant main effect of electrode location (*F*_[3,48]_ = 6.817, *p < *0.01). A pair-wise comparison confirmed that FRN at Fz was more negative (10.277 μV ± 1.109) than at other electrode locations. The main effect of feedback valence (*F*_[1,16]_ = 30.018, *p *< 0.001) was also significant, with losing decisions eliciting a more negative FRN (11.125 μV ± 1.162) than winning decisions (13.540 μV ± 1.207). Importantly, there was a significant interaction between valence and delay (*F*_[1,16]_ = 7.310,* p *< 0.05). A simple effect analysis showed that FRN amplitude was more negative for delayed gains (12.834 μV ± 1.193) than for immediate gains (14.247 μV ± 1.285; *F*_[1,16]_ = 6.20, *p *< 0.05), but no such difference was observed in the loss condition (*F*_[1,16]_ = 0.55, *p *= 0.471).

To further investigate this interaction effect, ERP amplitude of difference waves were analyzed on the four electrode locations. A paired samples *t*-test showed that, at Fz, the difference wave amplitude after immediate outcomes was significantly different from zero (-3.745 μV ± 2.470, *t*_(16)_ = -6.236, *p *< 0.001); the FRN effect following delay outcomes was also significant (-1.944 μV ± 2.012, *t*_(16)_ = -3.984,* p *< 0.01); and the sizes of the two difference waves were significantly different from each other (*t*_(16)_ = -2.947, *p *< 0.01). The FRN effect was more remarkable for immediate outcomes, and delay reduced the valence effect of FRN. Similar results were obtained when we analyzed data from FCz(-3.760 μV ± 2.578, *t*_(16)_ = -6.013, *p *< 0.001; -1.818 μV ± 1.945, *t*_(16)_ = -3.853, *p *< 0.01;* t*_(16)_ = -3.100, *p *< 0.01) and Cz(-3.550 μV ± 2.913, *t*_(16)_ = -5.025, *p *< 0.001; -1.445 μV ± 1.990, *t*_(16)_ = -2.995, *p *< 0.01;* t*_(16)_ = -2.825, *p *< 0.05).

To confirm that the amplitude of the FRN was not confounded by overlap with the P300, we followed the method of Holroyd and Krigolson (2007) to carry out paired samples *t-*tests on the amplitude of difference wave at Fz and Pz (where the peak of the P300 is usually localized). The result indicated that the difference waves for immediate outcomes were significantly larger at Fz than Pz (-3.735 μV vs. -2.169 μV, *t*_(16)_ = -4.652, *p* < 0.001). Similar results were obtained for delayed outcomes (-1.944 μV vs. -0.90 μV, *t*_(16)_ = -2.867, *p* < 0.05).

### P300

Similar analyzes were conducted for the peak values of the P300, which yielded a significant main effect of electrode location (*F*_[3,48]_ = 5.363 *p *< 0.01). Post-hoc analyzes showed a more positive P300 at Cz (18.904 μV ± 1.294) and Pz (18.310 μV ± 1.334) than that Fz (16.036 μV ± 1.086), *p* < 0.05. The main effect of valence was significant (*F*_[1,16]_ = 37.359, *p *< 0.001). The amplitude of P300 following gains (19.103 μV ± 1.185) was larger than that following losses (16.781 μV ± 1.142). There were no other significant effects (all *p*s > 0.239).

## DISCUSSION

Despite a wealth of research on temporal discounting, it is still poorly understood whether temporal information and valence could be encoded and integrated in the process of outcome evaluation as reflected by the FRN and P300 component. With the ERP technique, the present study employed a simple gambling task to investigate how temporal delay affects the brain activity in outcome evaluation. Valence and delay time were manipulated to explore how temporal information and valence were integrated and encoded in the FRN and P300. Four types of outcome were presented to participants: immediate gains, immediate losses, delayed gains, and delayed losses.

As expected, FRN was found to be larger in response to unfavorable outcomes. Regardless of time delay, losses were associated with a larger FRN than gains (Nieuwenhuis et al., 2004; Yeung and Sanfey, 2004; Holroyd et al., 2006; Hewig et al., 2007; Goyer et al., 2008). Interestingly, immediate loss and delayed loss elicited comparable FRNs, and delayed loss elicited a larger FRN than delayed gain. Moreover, delayed gain elicited a more negative FRN than immediate gain. This gradually decreasing amplitude of FRN suggests temporal information was integrated and reflected in FRN in the early stage of outcome evaluation. Furthermore, the graded coding of outcome in the FRN shed light on the neural basis of performance monitoring and outcome evaluation. Most previous researchers mainly focused on objective factors, such as the influence of the feedback valence, magnitude, and probability on the amplitude of the FRN (Yeung and Sanfey, 2004; Holroyd et al., 2006; Bellebaum et al., 2010; Kreussel et al., 2012). In recent years, some researchers have taken into account the subjective value of rewards by including social context and personality traits, such as social comparison (Boksem et al., 2011), interpersonal relationship (Fukushima and Hiraki, 2006, 2009; Itagaki and Katayama, 2008; Kang et al., 2010; Ma et al., 2011) and anxiety (Gu et al., 2010a,b). This previous research did indicate that the subjective value of the outcome could modulate FRN in response to our own or others’ performance or monetary outcomes.

Our findings provide further evidence that the subjective value rather than the objective value was encoded by ACC (indexed by the FRN) at an early stage. According to the motivational/affective hypothesis of the FRN (Gehring and Willoughby, 2002; Masaki et al., 2006), FRN reflects the motivational/affective significance of outcomes. If the FRN only encodes objective value, FRN would not be sensitive to time delay. However, we did observe a larger FRN elicited by delayed loss compared to delayed gain, whose amplitude was larger than that of immediate gain, which provided further evidence that reward was encoded by FRN in a fine-grained pattern according to the subjective value of outcome (Hajcak et al., 2007; Holroyd and Krigolson, 2007; Bellebaum and Daum, 2008; Bellebaum et al., 2010; Gu et al., 2011; Luo et al., 2011), rather than a binary pattern according to the good/bad objective value of outcome (Yeung and Sanfey, 2004; Toyomaki and Murohashi, 2005; Hajcak et al., 2006; Holroyd et al., 2006). The observed temporal delay effect is also consistent with previous neuroimaging studies in which the subjective value of delayed monetary rewards was tracked by reward processing areas of the brain, and delays of future rewards decreased activation in mesolimbic dopamine projection areas implicated in reward processing (Kable and Glimcher, 2007; Pine et al., 2009).

Moreover, the results demonstrated a greater FRN effect between gains and losses following immediate outcomes than that following delayed outcomes. The greater FRN effect in the immediate condition is in line with temporal discounting and the sign effect. This suggests the presence of temporal discounting, in which the subjective value of immediate rewards was larger than that of delayed rewards. In the gain conditions, immediate receipt is attractive and delaying receipt needs to be compensated. Thus, immediate gains are more preferable; whereas in loss conditions, immediate receipt is unattractive and people should be willing to pay a premium to put such events off, making delayed losses more preferable. According to model of sign effect, discounting rates for losses are typically far smaller than those for gains (Thaler, 1981). For example, delayed +10 RMB in the current experiment might be valued as +7 RMB immediate. As for future losses, delayed -10 RMB might be valued as -9 RMB immediate. Consequently, the difference between immediate gain and immediate loss is 20, which is more than that between delayed gain and delayed loss (16). Therefore, a larger difference between the subjective value of gain and loss in the immediate condition caused a greater FRN effect.

It is worth noting that we did not observe a differential FRN in the loss condition. One possible reason is the notion of reward positivity. Holroyd et al. (2008) proposed that, rather than a negativity in response to losses, activity in the time range of the FRN may reflect an underlying positivity in response to rewards that is reduced or absent in response to losses. During recent years, more and more studies have found evidence for this notion (Eppinger et al., 2009; San Martin et al., 2010; Foti et al., 2011). Therefore, this hypothesis might help us to explain the existence of modulation associated with gains, rather than losses. Another possible reason might be that the manipulation of time delay (1 month) in the current study is too short, as sign effect predicts a smaller difference between immediate loss and delayed loss, which might not be enough to cause a difference reflected in FRN. More specifically, given a longer time delay, the difference between immediate loss and delayed loss would be larger. Future studies will be needed to verify whether FRN could distinguish immediate losses from delayed losses when the delay time was manipulated gradually.

P300 was found to be modulated by valence, with a larger P300 in response to gains, which replicated previous studies (Hajcak et al., 2005, 2007; Wu and Zhou, 2009). Given that the P300 is widely believed to be related to processes of attentional allocation and to high-level motivational/affective evaluation (Yeung and Sanfey, 2004; Nieuwenhuis et al., 2005), it is possible that more attentional resources (Gray et al., 2004; Linden, 2005) are devoted to outcomes that benefit oneself.

To conclude, the present results are the first demonstration that temporal discounting and sign effect could be encoded in the FRN in the early stage of outcome evaluation, which add important neuroscience evidence of temporal discounting and deepen our understanding of outcome evaluation. The integration of valence and temporal information, which was reflected by FRN, also suggests that FRN works in a graded pattern with regard to subjective value of outcome, rather than a dichotomous pattern with regard to objective value of outcome. Future studies would investigate whether FRN could distinguish immediate losses from delayed losses when the delay time was manipulated gradually.

## Conflict of Interest Statement

The authors declare that the research was conducted in the absence of any commercial or financial relationships that could be construed as a potential conflict of interest.
